# Porous nickel doped titanium dioxide nanoparticles with improved visible light photocatalytic activity

**DOI:** 10.1039/c9na00760a

**Published:** 2020-02-29

**Authors:** Bingbing Guan, Jie Yu, Siyao Guo, Shen Yu, Song Han

**Affiliations:** College of Forestry, Northeast Forestry University Harbin 150040 China songh77@126.com; School of Civil Engineering, Qingdao University of Technology Qingdao 266033 China

## Abstract

A green hydrothermal synthesis route to prepare a porous nickel doped titanium dioxide (Ni–TiO_2_) nanostructured photocatalyst has been developed in this research. The results show that Ni doping can greatly increase the visible light photocatalytic performance of TiO_2_ through the introduction of impurity bands in the band gap of TiO_2_. After 5 cycles of reuse, Ni–TiO_2_ nanoparticles still show stable photocatalytic activity for MB degradation. The Ni–TiO_2_ nanoparticles developed in the present study are expected to have great potential applications in wastewater treatment due to the advantages of strong visible light photocatalytic performance, a simple synthetic process and high cycle utilization performance.

## Abstract 2

A green hydrothermal synthesis route to prepare a porous Ni doped TiO_2_ nanostructured photocatalyst has been developed in this research. Ni doping can increase the visible light photocatalytic performance of TiO_2_ with cycle capability of 5 times.

## Graphical abstract

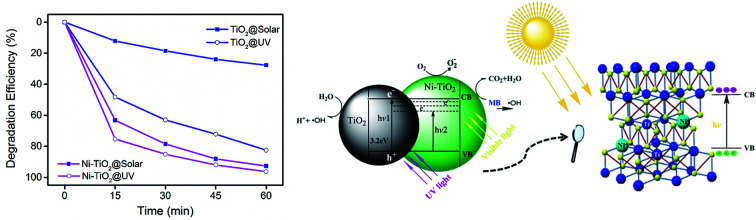

## 1. Introduction

Due to the prosperity of modern industries, especially the ones dealing with plastics, paper and textile dying, a huge amount of wastewater with various kinds of effluents is discharged, resulting in a great crisis in the acquirement of fresh water.^1^ Organic dye pollutants, one of the main produced effluents, can seriously disturb and destroy the ecological balance, leaving a heavy negative impact on the living, both human beings and plants.^2^ To mitigate the above mentioned crisis, a great number of studies have focused on dye wastewater treatment, and various strategies have been developed, such as biodegradation,^3^ chlorination,^4^ electrochemical,^5^ photocatalytic^6–10^ and adsorption^11,12^ methods. As one of the most effective methods, heterogeneous photocatalysis can greatly facilitate the oxidation of the pollutants and the by-products of hazardous organic pollutants.^12^ These catalysts typically have an excellent capability to convert photon energy into chemical energy which is favorable for the decomposition of the main toxic organic contaminants. Among these catalysts, TiO_2_ has been proved to be the most effective one due to its first usage in heterogeneous photocatalysis under UV light irradiation by Fujishima and Honda in 1972.^13^ Afterwards, photocatalysis with TiO_2_ catalysts became a research hotspot to decay the harmful chemical effluents present in wastewater.^14,15^ After several decades of development, anatase TiO_2_ is now considered to be one of the most common photocatalysts with high photocatalytic activity.^16^

TiO_2_ has a favorable band gap, good chemical stability, good photostability, and high corrosion resistance.^17,18^ TiO_2_ is also one of the most noticeable photocatalysts with particular properties: it is a recoverable and reusable catalyst and can offer an eco-friendly and non-toxic approach for dye wastewater treatment. The photocatalytic activity of TiO_2_ is based on the mechanism of the formation of electron/hole (e^−^/h^+^) pairs under the illumination of light which can initiate chemical reactions by generating radical species on the surface of TiO_2_.^19^ However, its poor efficiency in response to visible light limits its application due to the hindrance of the large band gap to catalyst efficiency under natural sunlight illumination which mostly contains visible regions.^20^

Doping of TiO_2_ with different transition metals (Fe, Mn, Cu, Ni, *etc.*) can enhance the degradation under visible light irradiation, which has been successfully applied in wastewater treatment.^21^ The reducing of the band gap of the catalysts has been achieved by doping metals through different processes. Benjwal *et al.* reported that a Zn and Mn co-doped TiO_2_ photocatalyst showed high activity and excellent adsorption properties in low concentration aqueous solutions.^22^ Copper phthalocyanine (CuPc) doped TiO_2_ was confirmed to be an efficient and stable photocatalyst for degradation of methylene blue from aqueous solution under solar light irradiation.^23^ The doping of TiO_2_ with other transition metals such as Fe, Ni and Co has also been employed in various applications.^24–26^ However, the applications of doped TiO_2_ are still limited by their high cost and relatively low stability.

To obtain highly effective TiO_2_ photocatalysts, the synthesis techniques need to be well controlled. In this work, novel Ni–TiO_2_ nanoparticles have been developed using a green hydrothermal-synthesis route. Different from traditional TiO_2_ preparation techniques, this synthesis route is easy to be operated and could save time. Meanwhile, the novel Ni–TiO_2_ nanoparticles exhibit outstanding performance on adsorption of MB dye from aqueous solutions in darkness and high photocatalytic activity towards MB dye under visible light. The catalyst also exhibits extremely high cycle performance and recyclability. The synthesis strategy presented in this work can prepare materials with outstanding properties and will show potential application in water treatment systems.

## 2. Experimental

### 2.1 Materials

Butyl titanate ([CH_3_(CH_2_)_3_O]_4_Ti), absolute ethyl alcohol (C_2_H_5_OH), hydrochloric acid (HCl), ammonium hydroxide (NH_3_·H_2_O), nickel nitrate (Ni(NO_3_)_2_·6H_2_O), and methylene blue (MB), were all purchased from Sinopharm Reagent Co Ltd. All the chemicals were of analytical grade and used without further purification. Deionized water was used throughout for the preparation of all the experimental solutions.

### 2.2 Preparation of TiO_2_ and Ni–TiO_2_ nanoparticles

Tetrabutyl titanate (10 mL) and absolute ethyl alcohol (10 mL) were mixed to obtain solution A. Absolute ethyl alcohol (20 mL) and deionized water (100 mL) were mixed to obtain solution B. Solution A was then added into solution B dropwise under magnetic stirring for 30 min. Then, the pH value was adjusted to 9 by ammonium hydroxide. After homogenization for 30 min, the mixed solution was transferred into a Teflon-lined autoclave for crystallization at 140 °C for 4 h. The resulting product was washed with ethyl alcohol and deionized water 3 times each. Then the nanoparticles were separated from the liquid phase by centrifugation to remove the remaining compounds. The final product was dried at 80 °C overnight to obtain TiO_2_ powders. The synthetic steps for Ni-(3 wt%) TiO_2_ nanoparticles were little different from the above. In step three, solution A and a nickel nitrate solution (0.85 mL, 1 mol L^−1^) were added into solution B dropwise under magnetic stirring for 30 min.

### 2.3 Characterization

FT-IR spectra were recorded using a Shimadzu instrument (model 8400S) in the region 4000–400 cm^−1^. The phase analysis of the as-synthesized products was carried out using X-ray diffraction (XRD, DX-2700) with Cu-Kα radiation (*λ* = 1.5406 Å). UV-vis-NIR absorption spectra of the samples were recorded using a UV-1800 spectrophotometer (Shimadzu). SEM images were obtained using a S-4800 instrument (Hitachi, Japan). The specific surface area was calculated by the Brunauer–Emmett–Teller (BET) method, and the pore size distribution was obtained using the Barrett–Joyner–Halenda (BJH) model using a Micromeritics ASAP 2020 adsorption analyzer.

### 2.4. Photocatalytic experiments

Degradation of MB was used as an indicator for the photocatalytic activity of the TiO_2_ nanoparticles. The prepared TiO_2_ nanoparticles were immersed in 10 mg L^−1^ MB solution and were allowed to completely equilibrate with MB for 20 min in darkness. Then the system was irradiated by simulated solar light (Xe lamp, 300 W) or UV light. 10 mL of solution was taken and analysed at different reaction times (every 15 min) using a UV-1800 spectrophotometer.

## 3. Results and discussion

### 3.1 FTIR spectra

**Fig. 1:**
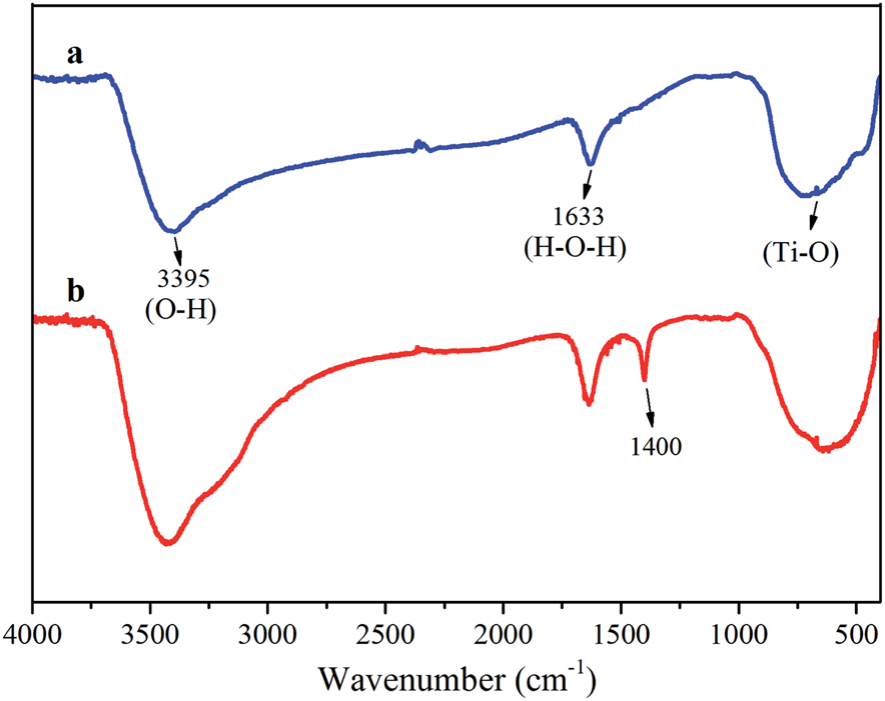
FT-IR spectral analysis of (a) TiO_2_ and (b) Ni–TiO_2_ nanoparticles.

Fig. 1 shows the FTIR spectra of the TiO_2_ and Ni–TiO_2_ nanoparticles. The strong absorption bands at 662 and 704 cm^−1^ might be due to the Ti–O vibrations in the TiO_2_ lattice. Furthermore, a broad absorption band in the region of 3000–3500 cm^−1^ can be assigned to the surface-bound hydroxyl groups and their stretching vibration on the surface of TiO_2_.^27^ A second adsorption band at 1000–1700 cm^−1^ is assigned to surface-adsorbed water molecules (H–O–H bending, 1633 cm^−1^).^28^ It can confirm a strong interaction of water molecules with the TiO_2_ surface to form a number of broad OH– stretching vibrations.^22^ A broad intense vibration region at 1000–1200 cm^−1^ is credited to the Ti–O–Ti vibration. Moreover, occurrence of bands between 1300–1500 cm^−1^ for Ni–TiO_2_ nanoparticles indicates the presence of a small amount of organic material in the sample.^29^ With an increase in Ni concentration, the shift to lower wavenumbers of the Ti–O–Ti band could be attributed to the increase in powder particle size.^30^

### 3.2 Phase analysis and morphology

**Fig. 2:**
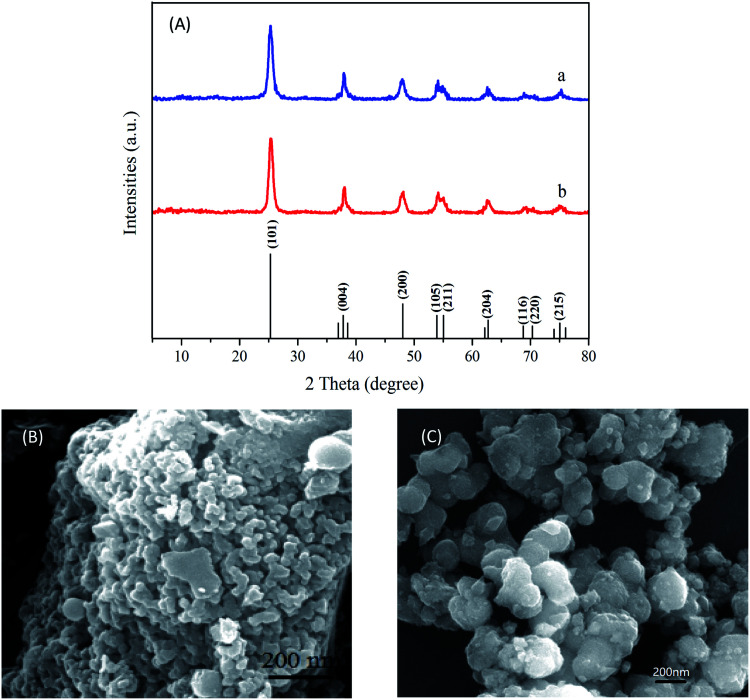
(A) XRD patterns of (a) TiO_2_ and (b) Ni–TiO_2_ nanoparticles; (B) morphology of Ni–TiO_2_; (C) morphology of TiO_2_.

XRD patterns of TiO_2_ and Ni–TiO_2_ nanoparticles are shown in Fig. 2A. The Ni–TiO_2_ sample exhibits peaks at 25.28°, 37.80°, 48.05°, 53.89°, 55.06°, 62.69°, 70.31°, and 75.03°, corresponding to the anatase phase (JCPDF 21-1272), with no other phases. In addition, peaks corresponding to Ni oxides are not detected. These results further indicate that Ni ions have been successfully doped into TiO_2_ nanoparticles.^2^Fig. 2B shows the spherically shaped Ni doped TiO_2_ nanostructures. Compared with the pure TiO_2_ image (Fig. 2C), Ni–TiO_2_ shows homogeneous nanoparticles with sizes of 20–30 nm.

### 3.3 UV-vis spectral analysis

**Fig. 3:**
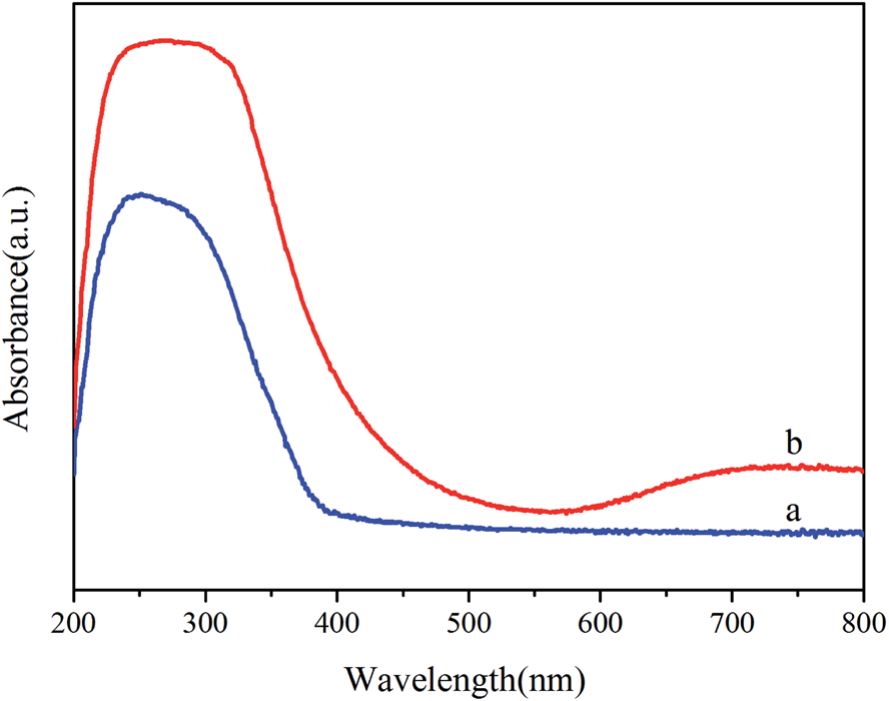
UV-visible absorption spectra of (a) TiO_2_ and (b) Ni–TiO_2_ samples.

The electronic structure of the samples that furnishes the optical properties (*e.g.*, absorption and band gap) through the irradiating light intensity was determined by UV-vis spectral analysis,^17^ as shown in Fig. 3. In the absorption spectra, it is noticeable that the optical absorption edge of the pure TiO_2_ is at 400 nm. The band gap of TiO_2_ is 3.21 eV which is favorable to produce electron–hole pairs under the UV light irradiation. However, the pure TiO_2_ can not degrade dyes under visible light. Compared to pure TiO_2_, Ni–TiO_2_ nanoparticles exhibit a broad absorption covering the range as shown in Fig. 3b, which can be ascribed to the doping energy levels caused by the doped Ni in the band gap of TiO_2_.

### 3.4 Nitrogen adsorption–desorption isotherm of Ni–TiO_2_

**Fig. 4:**
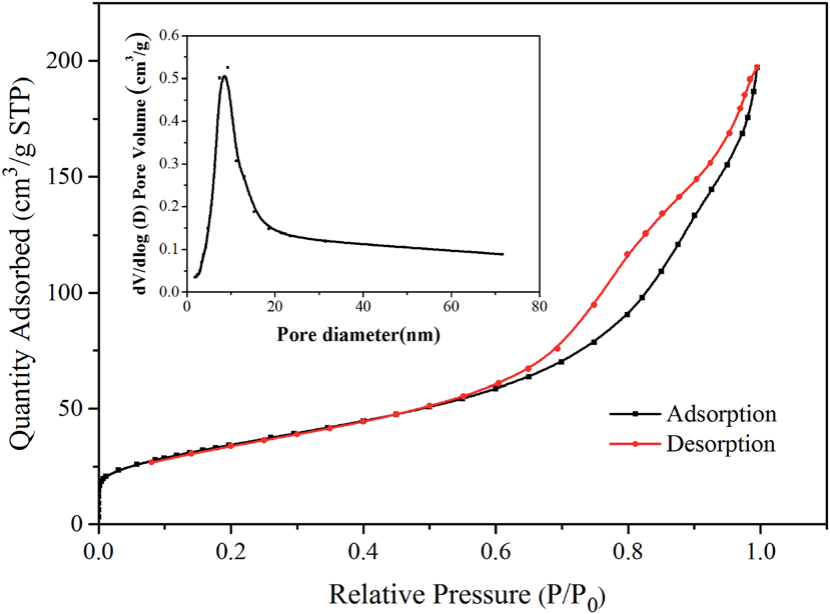
Nitrogen adsorption–desorption isotherms and pore size distribution curve of Ni–TiO_2_.

The nitrogen adsorption–desorption isotherm and BJH pore size distribution curve (inset) of Ni–TiO_2_ are shown in Fig. 4, which displays a type-IV isotherm with a specific surface area of 124.02 m^2^ g^−1^. This implies that the pores within the materials are mainly within the mesoporous range. The pore size distribution is calculated using the BJH method (desorption curve).^31^ The pore-size distribution of Ni–TiO_2_ shows that the pore diameters distribution (Fig. 4 inset) has a peak at about 9 nm, indicating that Ni–TiO_2_ has a mesoporous structure. These small pores can enhance photocatalytic activities by favoring the adsorption of small dye molecules on the active surface.

### 3.5 MB decomposition capacity under solar and UV light

**Fig. 5:**
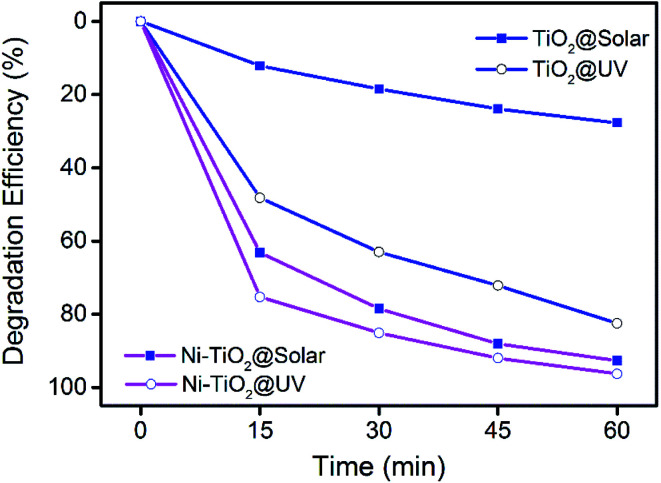
Photodegradation of MB dye using TiO_2_ and Ni–TiO_2_ nanoparticles under UV and solar light irradiation.

Fig. 5 depicts the photocatalytic degradation of MB using TiO_2_ and Ni–TiO_2_ nanoparticles under UV and solar light irradiation as a function of time with an initial MB concentration of 10 mg L^−1^. The degradation efficiencies of Ni–TiO_2_ nanoparticles under solar and UV after 60 min irradiation are found to be 92.7% and 96.3%, respectively. However, the corresponding degradation efficiencies are only 85.9% and 27.7% for pure TiO_2_. It can be found that Ni doping can increase the visible light degradation performance greatly. The TiO_2_ catalyst shows very weak photocatalytic performance for MB degradation under solar light irradiation. After Ni doping, the visible light photocatalytic performance increases to a similar level compared with that under UV light. The result demonstrates that Ni doping can improve the photocatalytic activity of TiO_2_ nanoparticles under visible light.

### 3.6 Photocatalytic performance stability

**Fig. 6:**
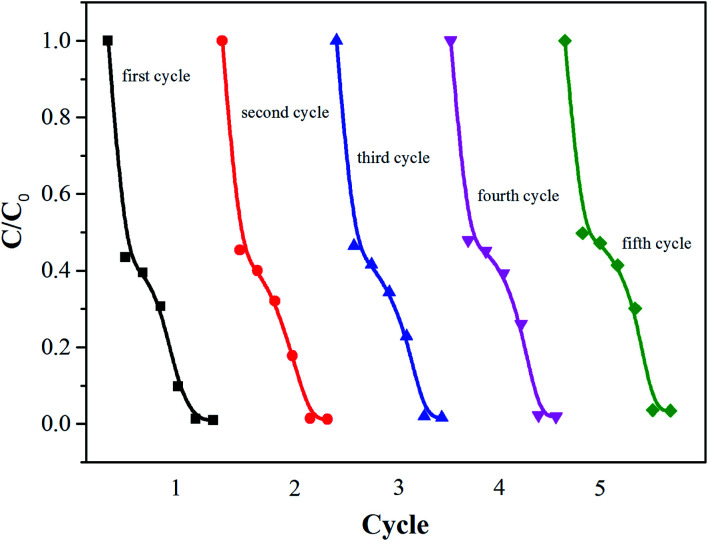
Photocatalytic stability test of Ni–TiO_2_ nanoparticles.

The stability is also important for the practical application of the photocatalyst. Therefore, the cyclic stability of Ni–TiO_2_ nanoparticles was investigated by monitoring the catalytic activity during successive cycles of degradation. As shown in Fig. 6, after a five cycles test, the Ni–TiO_2_ nanoparticles exhibit a very stable photocatalytic performance without any significant deactivation, thereby demonstrating high stability after multiple reuse cycles.

### 3.7 Photocatalytic mechanism of Ni–TiO_2_

**Fig. 7:**
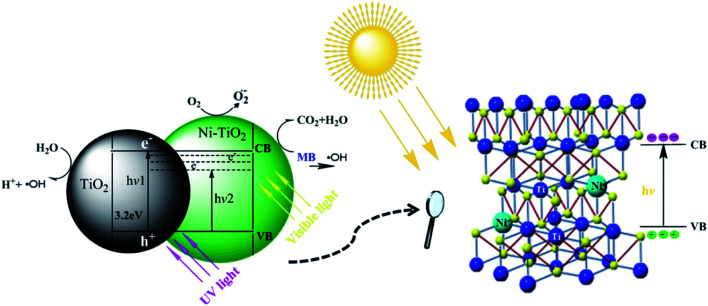
Possible mechanism of the MB degradation by Ni–TiO_2_ nanoparticles.

The plausible mechanism of the photocatalytic activity of the synthesized Ni–TiO_2_ nanoparticles can be explained by the energy band gap structure of TiO_2_ shown in Fig. 7. The direct excitation of an electron from the valence band (VB) to the conduction band (CB) in the presence of visible light is not possible due to the broad band gap (3.21 eV) of pure TiO_2_. Through the incorporation of Ni ions into the TiO_2_ lattice, the band gap of TiO_2_ decreases due to the formation of impurity levels below the CB in the band gap, then the electrons can transfer from the VB to these energy levels. These electrons travel to the surface and are adsorbed by O_2_ and produce ˙O_2_ ions, which can further convert to the strong redox species ˙OH ions.^32^ These redox ions are responsible for the degradation of the surface adsorbed hazardous MB.^22^

## 4. Conclusions

Ni–TiO_2_ nanoparticles were synthesized by a green hydrothermal-synthesis route and characterized in detail. The activities of the synthesized nanoparticles were studied through MB photocatalytic degradation. The results demonstrate that Ni doping can greatly increase the visible light photocatalytic performance of TiO_2_ through the introduction of impurity bands in the band gap of TiO_2_. After 5 cycles of reuse, Ni–TiO_2_ nanoparticles still show stable photocatalytic activity for MB degradation. Thus the Ni–TiO_2_ nanoparticles developed in the present study are expected to have great potential applications in wastewater treatment due to the advantages of strong visible light photocatalytic performance, a simple synthetic process and high cycle utilization performance.

## Conflicts of interest

There are no conflicts to declare.

## Supplementary Material
